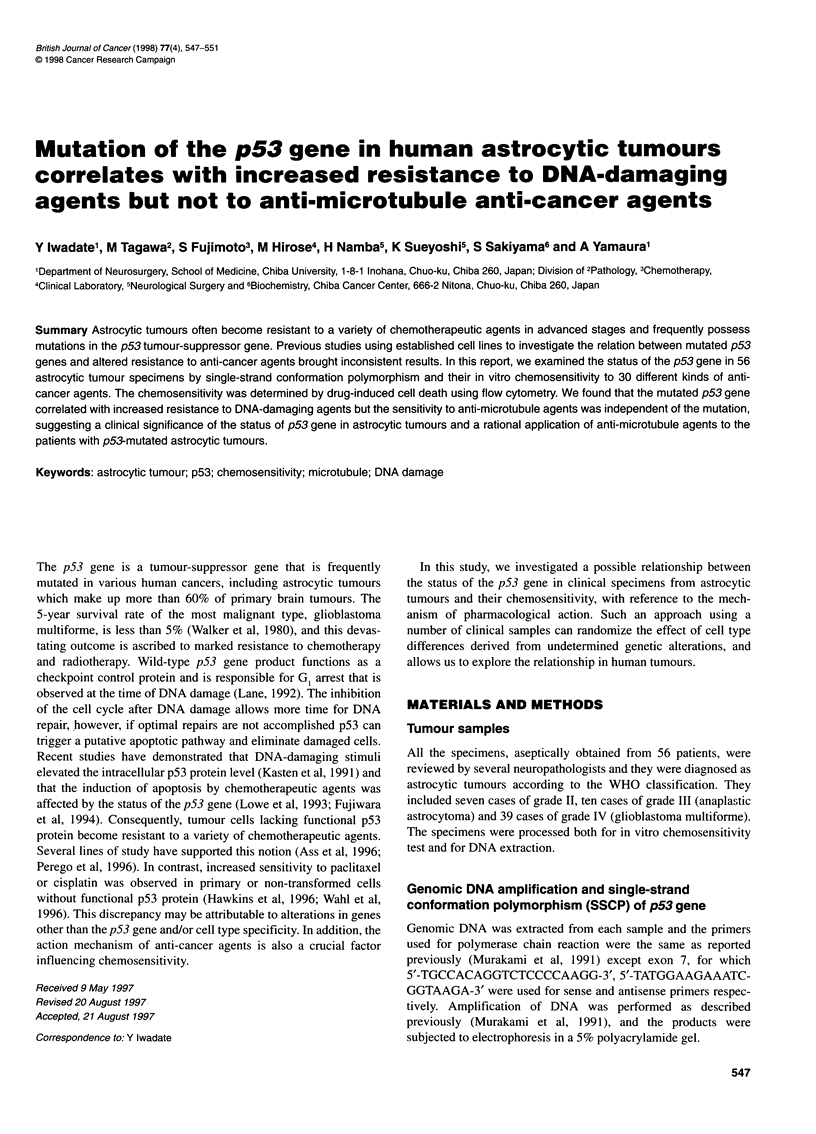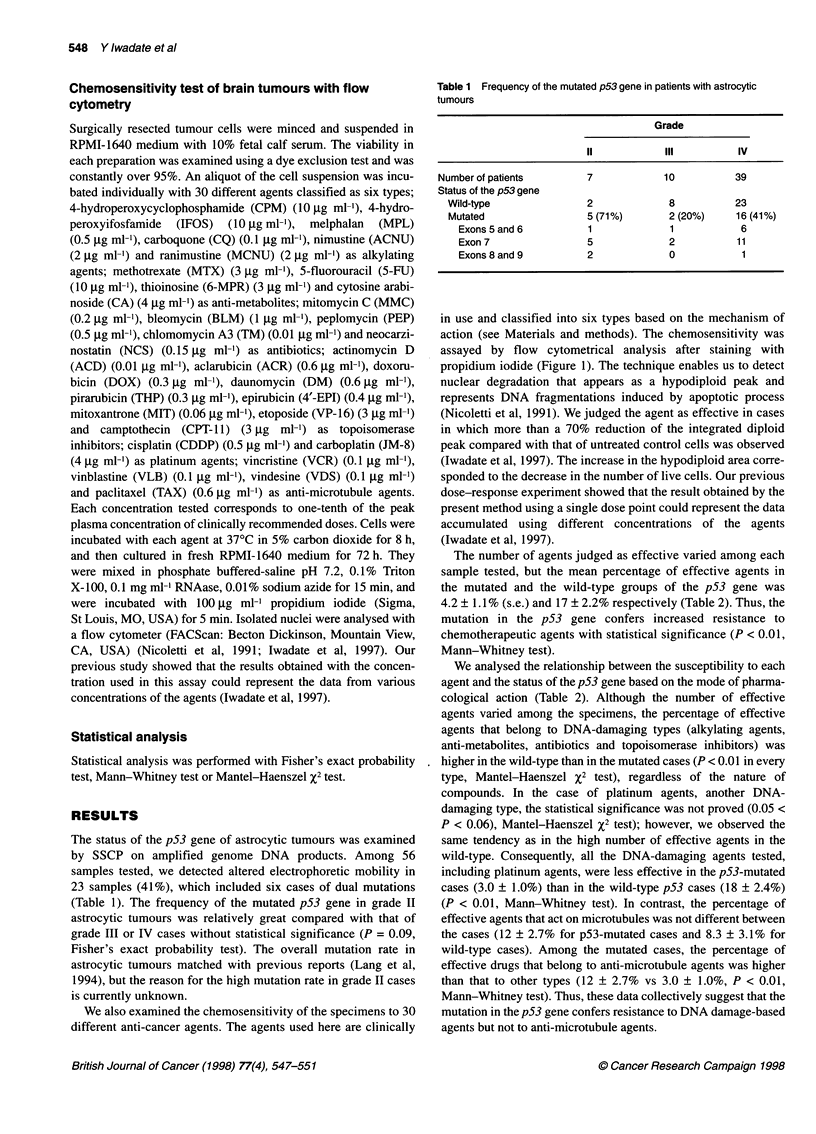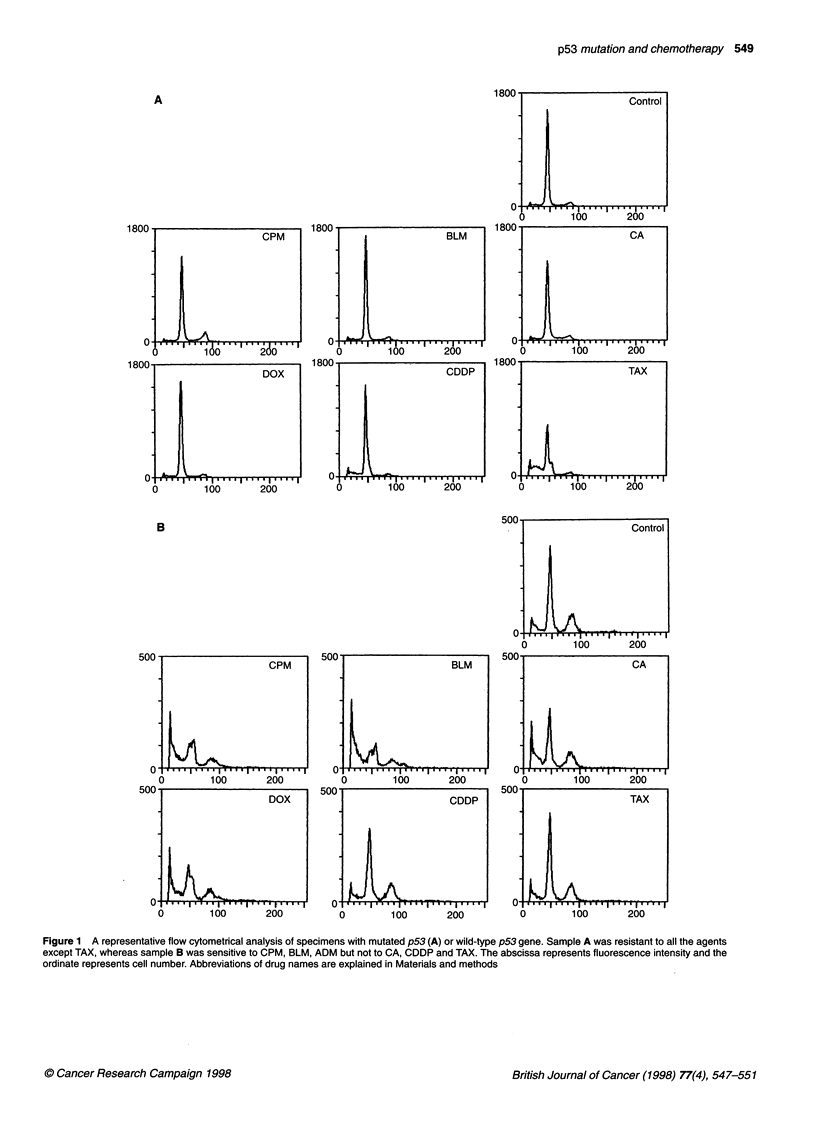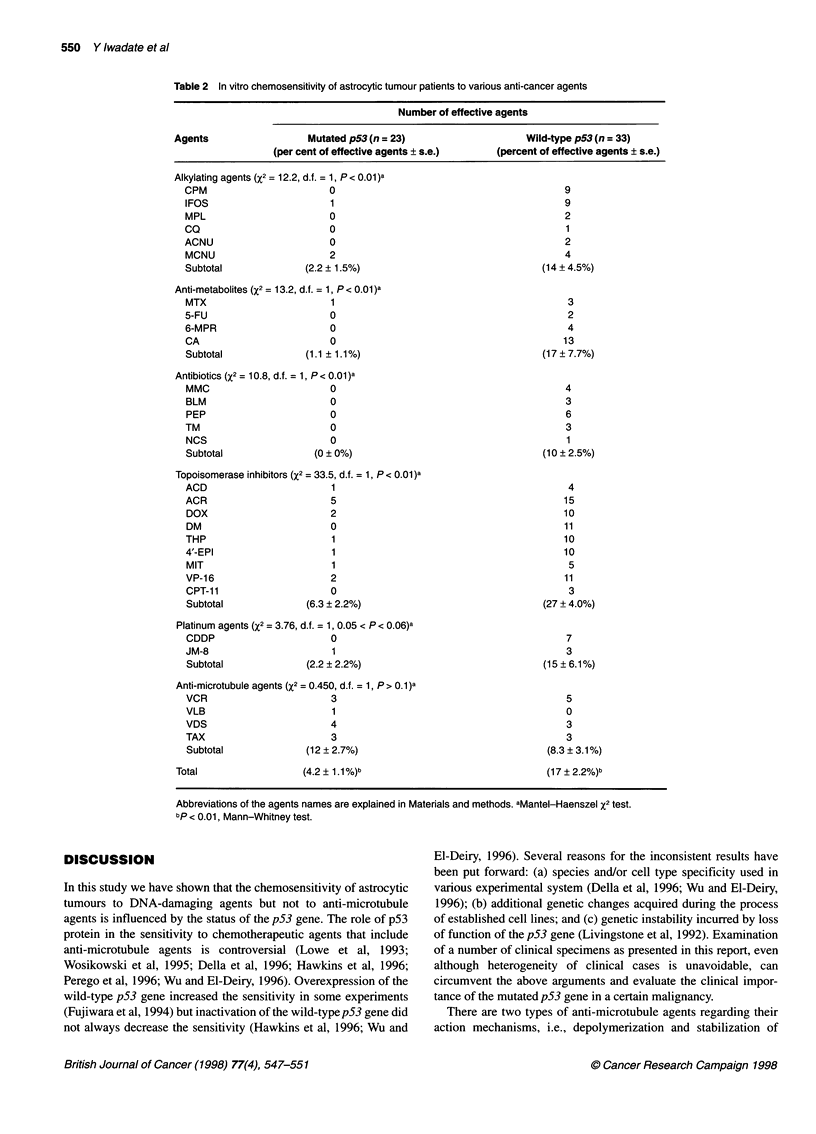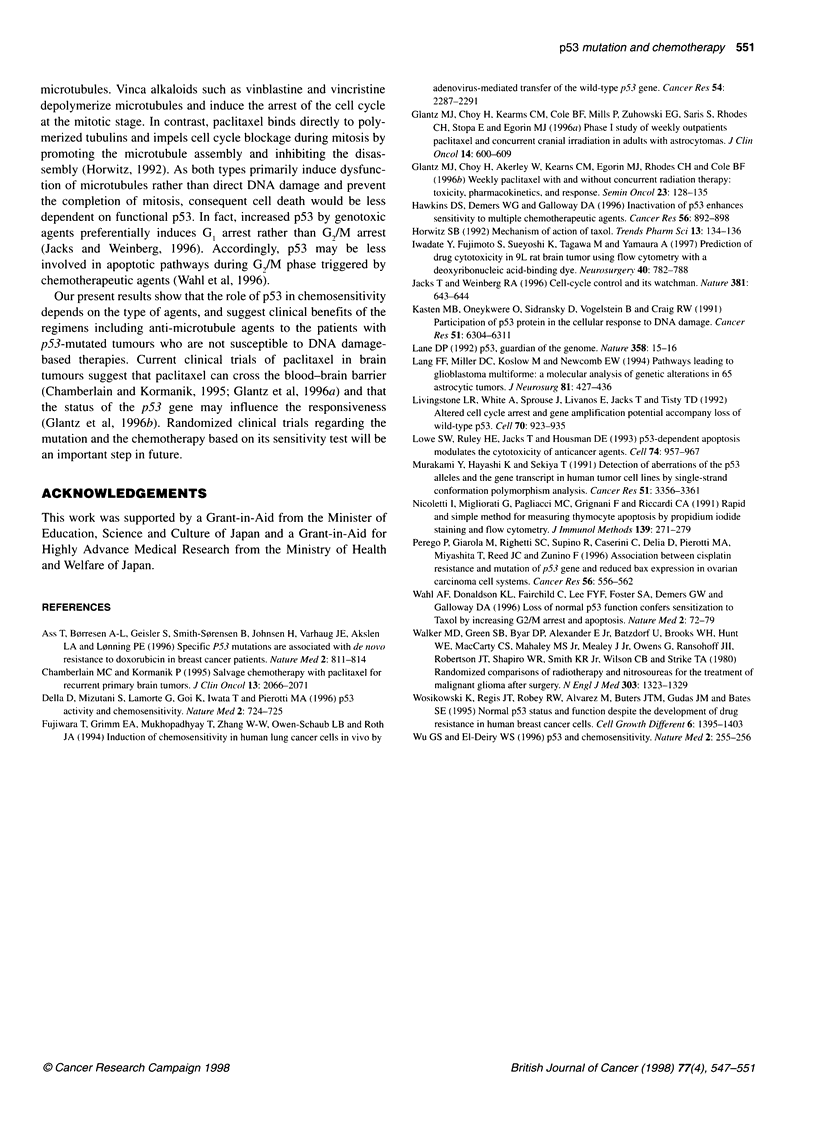# Mutation of the p53 gene in human astrocytic tumours correlates with increased resistance to DNA-damaging agents but not to anti-microtubule anti-cancer agents.

**DOI:** 10.1038/bjc.1998.88

**Published:** 1998-02

**Authors:** Y. Iwadate, M. Tagawa, S. Fujimoto, M. Hirose, H. Namba, K. Sueyoshi, S. Sakiyama, A. Yamaura

**Affiliations:** Department of Neurosurgery, School of Medicine, Chiba University, Japan.

## Abstract

Astrocytic tumours often become resistant to a variety of chemotherapeutic agents in advanced stages and frequently possess mutations in the p53 tumour-suppressor gene. Previous studies using established cell lines to investigate the relation between mutated p53 genes and altered resistance to anti-cancer agents brought inconsistent results. In this report, we examined the status of the p53 gene in 56 astrocytic tumour specimens by single-strand conformation polymorphism and their in vitro chemosensitivity to 30 different kinds of anti-cancer agents. The chemosensitivity was determined by drug-induced cell death using flow cytometry. We found that the mutated p53 gene correlated with increased resistance to DNA-damaging agents but the sensitivity to anti-microtubule agents was independent of the mutation, suggesting a clinical significance of the status of p53 gene in astrocytic tumours and a rational application of anti-microtubule agents to the patients with p53-mutated astrocytic tumours.


British Journal of Cancer (1998) 77(4), 547–551
© 1998 Cancer Research Campaign

### Mutation of the *p53* gene in human astrocytic tumours correlates with increased resistance to DNA-damaging agents but not to anti-microtubule anti-cancer agents

**Y Iwadate[1], M Tagawa[2], S Fujimoto[3], M Hirose[4], H Namba[5], K Sueyoshi[5], S Sakiyama[6] and A Yamaura[1]**

[1]Department of Neurosurgery, School of Medicine, Chiba University, 1-8-1 Inohana, Chuo-ku, Chiba 260, Japan; Division of [2]Pathology, [3]Chemotherapy, [4]Clinical Laboratory, [5]Neurological Surgery and [6]Biochemistry, Chiba Cancer Center, 666-2 Nitona, Chuo-ku, Chiba 260, Japan

**Summary** Astrocytic tumours often become resistant to a variety of chemotherapeutic agents in advanced stages and frequently possess mutations in the *p53* tumour-suppressor gene. Previous studies using established cell lines to investigate the relation between mutated *p53* genes and altered resistance to anti-cancer agents brought inconsistent results. In this report, we examined the status of the *p53* gene in 56 astrocytic tumour specimens by single-strand conformation polymorphism and their in vitro chemosensitivity to 30 different kinds of anti-cancer agents. The chemosensitivity was determined by drug-induced cell death using flow cytometry. We found that the mutated *p53* gene correlated with increased resistance to DNA-damaging agents but the sensitivity to anti-microtubule agents was independent of the mutation, suggesting a clinical significance of the status of *p53* gene in astrocytic tumours and a rational application of anti-microtubule agents to the patients with *p53*-mutated astrocytic tumours.

**Keywords**: astrocytic tumour; p53; chemosensitivity; microtubule; DNA damage

The *p53* gene is a tumour-suppressor gene that is frequently mutated in various human cancers, including astrocytic tumours which make up more than 60% of primary brain tumours. The 5-year survival rate of the most malignant type, glioblastoma multiforme, is less than 5% (Walker et al, 1980), and this devastating outcome is ascribed to marked resistance to chemotherapy and radiotherapy. Wild-type *p53* gene product functions as a checkpoint control protein and is responsible for $G_1$ arrest that is observed at the time of DNA damage (Lane, 1992). The inhibition of the cell cycle after DNA damage allows more time for DNA repair, however, if optimal repairs are not accomplished p53 can trigger a putative apoptotic pathway and eliminate damaged cells. Recent studies have demonstrated that DNA-damaging stimuli elevated the intracellular p53 protein level (Kasten et al, 1991) and that the induction of apoptosis by chemotherapeutic agents was affected by the status of the *p53* gene (Lowe et al, 1993; Fujiwara et al, 1994). Consequently, tumour cells lacking functional p53 protein become resistant to a variety of chemotherapeutic agents. Several lines of study have supported this notion (Ass et al, 1996; Perego et al, 1996). In contrast, increased sensitivity to paclitaxel or cisplatin was observed in primary or non-transformed cells without functional p53 protein (Hawkins et al, 1996; Wahl et al, 1996). This discrepancy may be attributable to alterations in genes other than the *p53* gene and/or cell type specificity. In addition, the action mechanism of anti-cancer agents is also a crucial factor influencing chemosensitivity.

*Received 9 May 1997*
*Revised 20 August 1997*
*Accepted, 21 August 1997*

*Correspondence to:* Y Iwadate

In this study, we investigated a possible relationship between the status of the *p53* gene in clinical specimens from astrocytic tumours and their chemosensitivity, with reference to the mechanism of pharmacological action. Such an approach using a number of clinical samples can randomize the effect of cell type differences derived from undetermined genetic alterations, and allows us to explore the relationship in human tumours.

### MATERIALS AND METHODS

### Tumour samples

All the specimens, aseptically obtained from 56 patients, were reviewed by several neuropathologists and they were diagnosed as astrocytic tumours according to the WHO classification. They included seven cases of grade II, ten cases of grade III (anaplastic astrocytoma) and 39 cases of grade IV (glioblastoma multiforme). The specimens were processed both for in vitro chemosensitivity test and for DNA extraction.

### Genomic DNA amplification and single-strand conformation polymorphism (SSCP) of *p53* gene

Genomic DNA was extracted from each sample and the primers used for polymerase chain reaction were the same as reported previously (Murakami et al, 1991) except exon 7, for which 5′-TGCCACAGGTCTCCCCAAGG-3′, 5′-TATGGAAGAAATC-GGTAAGA-3′ were used for sense and antisense primers respectively. Amplification of DNA was performed as described previously (Murakami et al, 1991), and the products were subjected to electrophoresis in a 5% polyacrylamide gel.

### Chemosensitivity test of brain tumours with flow cytometry

Surgically resected tumour cells were minced and suspended in RPMI-1640 medium with 10% fetal calf serum. The viability in each preparation was examined using a dye exclusion test and was constantly over 95%. An aliquot of the cell suspension was incubated individually with 30 different agents classified as six types; 4-hydroperoxycyclophosphamide (CPM) (10 μg $ml^{-1}$), 4-hydroperoxyifosfamide (IFOS) (10 μg $ml^{-1}$), melphalan (MPL) (0.5 μg $ml^{-1}$), carboquone (CQ) (0.1 μg $ml^{-1}$), nimustine (ACNU) (2 μg $ml^{-1}$) and ranimustine (MCNU) (2 μg $ml^{-1}$) as alkylating agents; methotrexate (MTX) (3 μg $ml^{-1}$), 5-fluorouracil (5-FU) (10 μg $ml^{-1}$), thioinosine (6-MPR) (3 μg $ml^{-1}$) and cytosine arabinoside (CA) (4 μg $ml^{-1}$) as anti-metabolites; mitomycin C (MMC) (0.2 μg $ml^{-1}$), bleomycin (BLM) (1 μg $ml^{-1}$), peplomycin (PEP) (0.5 μg $ml^{-1}$), chlomomycin A3 (TM) (0.01 μg $ml^{-1}$) and neocarzinostatin (NCS) (0.15 μg $ml^{-1}$) as antibiotics; actinomycin D (ACD) (0.01 μg $ml^{-1}$), aclarubicin (ACR) (0.6 μg $ml^{-1}$), doxorubicin (DOX) (0.3 μg $ml^{-1}$), daunomycin (DM) (0.6 μg $ml^{-1}$), pirarubicin (THP) (0.3 μg $ml^{-1}$), epirubicin (4′-EPI) (0.4 μg $ml^{-1}$), mitoxantrone (MIT) (0.06 μg $ml^{-1}$), etoposide (VP-16) (3 μg $ml^{-1}$) and camptothecin (CPT-11) (3 μg $ml^{-1}$) as topoisomerase inhibitors; cisplatin (CDDP) (0.5 μg $ml^{-1}$) and carboplatin (JM-8) (4 μg $ml^{-1}$) as platinum agents; vincristine (VCR) (0.1 μg $ml^{-1}$), vinblastine (VLB) (0.1 μg $ml^{-1}$), vindesine (VDS) (0.1 μg $ml^{-1}$) and paclitaxel (TAX) (0.6 μg $ml^{-1}$) as anti-microtubule agents. Each concentration tested corresponds to one-tenth of the peak plasma concentration of clinically recommended doses. Cells were incubated with each agent at 37°C in 5% carbon dioxide for 8 h, and then cultured in fresh RPMI-1640 medium for 72 h. They were mixed in phosphate buffered-saline pH 7.2, 0.1% Triton X-100, 0.1 mg $ml^{-1}$ RNAase, 0.01% sodium azide for 15 min, and were incubated with 100 μg $ml^{-1}$ propidium iodide (Sigma, St Louis, MO, USA) for 5 min. Isolated nuclei were analysed with a flow cytometer (FACScan: Becton Dickinson, Mountain View, CA, USA) (Nicoletti et al, 1991; Iwadate et al, 1997). Our previous study showed that the results obtained with the concentration used in this assay could represent the data from various concentrations of the agents (Iwadate et al, 1997).

### Statistical analysis

Statistical analysis was performed with Fisher's exact probability test, Mann–Whitney test or Mantel–Haenszel $\chi^2$ test.

### RESULTS

The status of the *p53* gene of astrocytic tumours was examined by SSCP on amplified genome DNA products. Among 56 samples tested, we detected altered electrophoretic mobility in 23 samples (41%), which included six cases of dual mutations (Table 1). The frequency of the mutated *p53* gene in grade II astrocytic tumours was relatively great compared with that of grade III or IV cases without statistical significance ($P$ = 0.09, Fisher's exact probability test). The overall mutation rate in astrocytic tumours matched with previous reports (Lang et al, 1994), but the reason for the high mutation rate in grade II cases is currently unknown.

We also examined the chemosensitivity of the specimens to 30 different anti-cancer agents. The agents used here are clinically in use and classified into six types based on the mechanism of action (see Materials and methods). The chemosensitivity was assayed by flow cytometrical analysis after staining with propidium iodide (Figure 1). The technique enables us to detect nuclear degradation that appears as a hypodiploid peak and represents DNA fragmentations induced by apoptotic process (Nicoletti et al, 1991). We judged the agent as effective in cases in which more than a 70% reduction of the integrated diploid peak compared with that of untreated control cells was observed (Iwadate et al, 1997). The increase in the hypodiploid area corresponded to the decrease in the number of live cells. Our previous dose–response experiment showed that the result obtained by the present method using a single dose point could represent the data accumulated using different concentrations of the agents (Iwadate et al, 1997).

**Table 1** Frequency of the mutated *p53* gene in patients with astrocytic tumours

| | Grade | | |
|---|---|---|---|
| | II | III | IV |
| Number of patients | 7 | 10 | 39 |
| Status of the *p53* gene | | | |
| Wild-type | 2 | 8 | 23 |
| Mutated | 5 (71%) | 2 (20%) | 16 (41%) |
| Exons 5 and 6 | 1 | 1 | 6 |
| Exon 7 | 5 | 2 | 11 |
| Exons 8 and 9 | 2 | 0 | 1 |

The number of agents judged as effective varied among each sample tested, but the mean percentage of effective agents in the mutated and the wild-type groups of the *p53* gene was 4.2 ± 1.1% (s.e.) and 17 ± 2.2% respectively (Table 2). Thus, the mutation in the *p53* gene confers increased resistance to chemotherapeutic agents with statistical significance ($P < 0.01$, Mann–Whitney test).

We analysed the relationship between the susceptibility to each agent and the status of the *p53* gene based on the mode of pharmacological action (Table 2). Although the number of effective agents varied among the specimens, the percentage of effective agents that belong to DNA-damaging types (alkylating agents, anti-metabolites, antibiotics and topoisomerase inhibitors) was higher in the wild-type than in the mutated cases ($P < 0.01$ in every type, Mantel–Haenszel $\chi^2$ test), regardless of the nature of compounds. In the case of platinum agents, another DNA-damaging type, the statistical significance was not proved (0.05 < $P$ < 0.06), Mantel–Haenszel $\chi^2$ test); however, we observed the same tendency as in the high number of effective agents in the wild-type. Consequently, all the DNA-damaging agents tested, including platinum agents, were less effective in the *p53*-mutated cases (3.0 ± 1.0%) than in the wild-type *p53* cases (18 ± 2.4%) ($P < 0.01$, Mann–Whitney test). In contrast, the percentage of effective agents that act on microtubules was not different between the cases (12 ± 2.7% for p53-mutated cases and 8.3 ± 3.1% for wild-type cases). Among the mutated cases, the percentage of effective drugs that belong to anti-microtubule agents was higher than that to other types (12 ± 2.7% vs 3.0 ± 1.0%, $P < 0.01$, Mann–Whitney test). Thus, these data collectively suggest that the mutation in the *p53* gene confers resistance to DNA damage-based agents but not to anti-microtubule agents.

© Cancer Research Campaign 1998

**Figure 1** A representative flow cytometrical analysis of specimens with mutated *p53* (**A**) or wild-type *p53* gene. Sample **A** was resistant to all the agents except TAX, whereas sample **B** was sensitive to CPM, BLM, ADM but not to CA, CDDP and TAX. The abscissa represents fluorescence intensity and the ordinate represents cell number. Abbreviations of drug names are explained in Materials and methods

**Table 2** In vitro chemosensitivity of astrocytic tumour patients to various anti-cancer agents

| Agents | Number of effective agents: Mutated *p53* ($n$ = 23) (per cent of effective agents ± s.e.) | Wild-type *p53* ($n$ = 33) (percent of effective agents ± s.e.) |
|---|---|---|
| Alkylating agents ($\chi^2$ = 12.2, d.f. = 1, $P < 0.01$)[a] | | |
| CPM | 0 | 9 |
| IFOS | 1 | 9 |
| MPL | 0 | 2 |
| CQ | 0 | 1 |
| ACNU | 0 | 2 |
| MCNU | 2 | 4 |
| Subtotal | (2.2 ± 1.5%) | (14 ± 4.5%) |
| Anti-metabolites ($\chi^2$ = 13.2, d.f. = 1, $P < 0.01$)[a] | | |
| MTX | 1 | 3 |
| 5-FU | 0 | 2 |
| 6-MPR | 0 | 4 |
| CA | 0 | 13 |
| Subtotal | (1.1 ± 1.1%) | (17 ± 7.7%) |
| Antibiotics ($\chi^2$ = 10.8, d.f. = 1, $P < 0.01$)[a] | | |
| MMC | 0 | 4 |
| BLM | 0 | 3 |
| PEP | 0 | 6 |
| TM | 0 | 3 |
| NCS | 0 | 1 |
| Subtotal | (0 ± 0%) | (10 ± 2.5%) |
| Topoisomerase inhibitors ($\chi^2$ = 33.5, d.f. = 1, $P < 0.01$)[a] | | |
| ACD | 1 | 4 |
| ACR | 5 | 15 |
| DOX | 2 | 10 |
| DM | 0 | 11 |
| THP | 1 | 10 |
| 4′-EPI | 1 | 10 |
| MIT | 1 | 5 |
| VP-16 | 2 | 11 |
| CPT-11 | 0 | 3 |
| Subtotal | (6.3 ± 2.2%) | (27 ± 4.0%) |
| Platinum agents ($\chi^2$ = 3.76, d.f. = 1, $0.05 < P < 0.06$)[a] | | |
| CDDP | 0 | 7 |
| JM-8 | 1 | 3 |
| Subtotal | (2.2 ± 2.2%) | (15 ± 6.1%) |
| Anti-microtubule agents ($\chi^2$ = 0.450, d.f. = 1, $P > 0.1$)[a] | | |
| VCR | 3 | 5 |
| VLB | 1 | 0 |
| VDS | 4 | 3 |
| TAX | 3 | 3 |
| Subtotal | (12 ± 2.7%) | (8.3 ± 3.1%) |
| Total | (4.2 ± 1.1%)[b] | (17 ± 2.2%)[b] |

Abbreviations of the agents names are explained in Materials and methods. [a]Mantel–Haenszel $\chi^2$ test. [b]$P < 0.01$, Mann–Whitney test.

### DISCUSSION

In this study we have shown that the chemosensitivity of astrocytic tumours to DNA-damaging agents but not to anti-microtubule agents is influenced by the status of the *p53* gene. The role of p53 protein in the sensitivity to chemotherapeutic agents that include anti-microtubule agents is controversial (Lowe et al, 1993; Wosikowski et al, 1995; Della et al, 1996; Hawkins et al, 1996; Perego et al, 1996; Wu and El-Deiry, 1996). Overexpression of the wild-type *p53* gene increased the sensitivity in some experiments (Fujiwara et al, 1994) but inactivation of the wild-type *p53* gene did not always decrease the sensitivity (Hawkins et al, 1996; Wu and El-Deiry, 1996). Several reasons for the inconsistent results have been put forward: (a) species and/or cell type specificity used in various experimental system (Della et al, 1996; Wu and El-Deiry, 1996); (b) additional genetic changes acquired during the process of established cell lines; and (c) genetic instability incurred by loss of function of the *p53* gene (Livingstone et al, 1992). Examination of a number of clinical specimens as presented in this report, even although heterogeneity of clinical cases is unavoidable, can circumvent the above arguments and evaluate the clinical importance of the mutated *p53* gene in a certain malignancy.

There are two types of anti-microtubule agents regarding their action mechanisms, i.e., depolymerization and stabilization of

© Cancer Research Campaign 1998

microtubules. Vinca alkaloids such as vinblastine and vincristine depolymerize microtubules and induce the arrest of the cell cycle at the mitotic stage. In contrast, paclitaxel binds directly to polymerized tubulins and impels cell cycle blockage during mitosis by promoting the microtubule assembly and inhibiting the disassembly (Horwitz, 1992). As both types primarily induce dysfunction of microtubules rather than direct DNA damage and prevent the completion of mitosis, consequent cell death would be less dependent on functional p53. In fact, increased p53 by genotoxic agents preferentially induces $G_1$ arrest rather than $G_2$/M arrest (Jacks and Weinberg, 1996). Accordingly, p53 may be less involved in apoptotic pathways during $G_2$/M phase triggered by chemotherapeutic agents (Wahl et al, 1996).

Our present results show that the role of p53 in chemosensitivity depends on the type of agents, and suggest clinical benefits of the regimens including anti-microtubule agents to the patients with *p53*-mutated tumours who are not susceptible to DNA damage-based therapies. Current clinical trials of paclitaxel in brain tumours suggest that paclitaxel can cross the blood–brain barrier (Chamberlain and Kormanik, 1995; Glantz et al, 1996*a*) and that the status of the *p53* gene may influence the responsiveness (Glantz et al, 1996*b*). Randomized clinical trials regarding the mutation and the chemotherapy based on its sensitivity test will be an important step in future.

### ACKNOWLEDGEMENTS

This work was supported by a Grant-in-Aid from the Minister of Education, Science and Culture of Japan and a Grant-in-Aid for Highly Advance Medical Research from the Ministry of Health and Welfare of Japan.

### REFERENCES

Ass T, Børresen A-L, Geisler S, Smith-Sørensen B, Johnsen H, Varhaug JE, Akslen LA and Lønning PE (1996) Specific *P53* mutations are associated with *de novo* resistance to doxorubicin in breast cancer patients. *Nature Med* **2**: 811–814

Chamberlain MC and Kormanik P (1995) Salvage chemotherapy with paclitaxel for recurrent primary brain tumors. *J Clin Oncol* **13**: 2066–2071

Della D, Mizutani S, Lamorte G, Goi K, Iwata T and Pierotti MA (1996) p53 activity and chemosensitivity. *Nature Med* **2**: 724–725

Fujiwara T, Grimm EA, Mukhopadhyay T, Zhang W-W, Owen-Schaub LB and Roth JA (1994) Induction of chemosensitivity in human lung cancer cells in vivo by adenovirus-mediated transfer of the wild-type *p53* gene. *Cancer Res* **54**: 2287–2291

Glantz MJ, Choy H, Kearms CM, Cole BF, Mills P, Zuhowski EG, Saris S, Rhodes CH, Stopa E and Egorin MJ (1996*a*) Phase I study of weekly outpatients paclitaxel and concurrent cranial irradiation in adults with astrocytomas. *J Clin Oncol* **14**: 600–609

Glantz MJ, Choy H, Akerley W, Kearns CM, Egorin MJ, Rhodes CH and Cole BF (1996*b*) Weekly paclitaxel with and without concurrent radiation therapy: toxicity, pharmacokinetics, and response. *Semin Oncol* **23**: 128–135

Hawkins DS, Demers WG and Galloway DA (1996) Inactivation of p53 enhances sensitivity to multiple chemotherapeutic agents. *Cancer Res* **56**: 892–898

Horwitz SB (1992) Mechanism of action of taxol. *Trends Pharm Sci* **13**: 134–136

Iwadate Y, Fujimoto S, Sueyoshi K, Tagawa M and Yamaura A (1997) Prediction of drug cytotoxicity in 9L rat brain tumor using flow cytometry with a deoxyribonucleic acid-binding dye. *Neurosurgery* **40**: 782–788

Jacks T and Weinberg RA (1996) Cell-cycle control and its watchman. *Nature* **381**: 643–644

Kasten MB, Oneykwere O, Sidransky D, Vogelstein B and Craig RW (1991) Participation of p53 protein in the cellular response to DNA damage. *Cancer Res* **51**: 6304–6311

Lane DP (1992) p53, guardian of the genome. *Nature* **358**: 15–16

Lang FF, Miller DC, Koslow M and Newcomb EW (1994) Pathways leading to glioblastoma multiforme: a molecular analysis of genetic alterations in 65 astrocytic tumors. *J Neurosurg* **81**: 427–436

Livingstone LR, White A, Sprouse J, Livanos E, Jacks T and Tisty TD (1992) Altered cell cycle arrest and gene amplification potential accompany loss of wild-type p53. *Cell* **70**: 923–935

Lowe SW, Ruley HE, Jacks T and Housman DE (1993) p53-dependent apoptosis modulates the cytotoxicity of anticancer agents. *Cell* **74**: 957–967

Murakami Y, Hayashi K and Sekiya T (1991) Detection of aberrations of the p53 alleles and the gene transcript in human tumor cell lines by single-strand conformation polymorphism analysis. *Cancer Res* **51**: 3356–3361

Nicoletti I, Migliorati G, Pagliacci MC, Grignani F and Riccardi CA (1991) Rapid and simple method for measuring thymocyte apoptosis by propidium iodide staining and flow cytometry. *J Immunol Methods* **139**: 271–279

Perego P, Giarola M, Righetti SC, Supino R, Caserini C, Delia D, Pierotti MA, Miyashita T, Reed JC and Zunino F (1996) Association between cisplatin resistance and mutation of *p53* gene and reduced bax expression in ovarian carcinoma cell systems. *Cancer Res* **56**: 556–562

Wahl AF, Donaldson KL, Fairchild C, Lee FYF, Foster SA, Demers GW and Galloway DA (1996) Loss of normal p53 function confers sensitization to Taxol by increasing G2/M arrest and apoptosis. *Nature Med* **2**: 72–79

Walker MD, Green SB, Byar DP, Alexander E Jr, Batzdorf U, Brooks WH, Hunt WE, MacCarty CS, Mahaley MS Jr, Mealey J Jr, Owens G, Ransohoff JII, Robertson JT, Shapiro WR, Smith KR Jr, Wilson CB and Strike TA (1980) Randomized comparisons of radiotherapy and nitrosoureas for the treatment of malignant glioma after surgery. *N Engl J Med* **303**: 1323–1329

Wosikowski K, Regis JT, Robey RW, Alvarez M, Buters JTM, Gudas JM and Bates SE (1995) Normal p53 status and function despite the development of drug resistance in human breast cancer cells. *Cell Growth Different* **6**: 1395–1403

Wu GS and El-Deiry WS (1996) p53 and chemosensitivity. *Nature Med* **2**: 255–256

© Cancer Research Campaign 1998